# Validation of the Chinese Empowerment of Parents in the Intensive Care (EMPATHIC-30) Questionnaire Among Parents in Neonatal Intensive Care Units: A Prospective Cross-Sectional Study

**DOI:** 10.3389/fped.2022.851291

**Published:** 2022-03-30

**Authors:** Yan Zhuang, Rong Zhang, Xi-rong Gao, Li-hui Zhu, Jos M. Latour

**Affiliations:** ^1^Neonatal Department, Hunan Children's Hospital, Changsha, China; ^2^Nursing Department, Hunan Children's Hospital, Changsha, China; ^3^Faculty of Health, University of Plymouth, Plymouth, United Kingdom

**Keywords:** neonatology, parents, infants, patient satisfaction, Neonatal Intensive Care Unit, EMPATHIC-30, reliability, validity

## Abstract

**Background:**

There are no specific validated questionnaires assessing satisfaction of family-centered care experienced by parents of infants hospitalized in Chinese Neonatal Intensive Care Units (NICU).

**Aim:**

To adapt and test the reliability and validity of the Chinese version of the EMPATHIC-30 questionnaire in NICU settings.

**Methods:**

A prospective, cross-sectional design was adopted. The EMPATHIC-30 questionnaires were completed by parents of infants admitted to one of the four NICUs at Hunan Children's Hospital, China, between November 2018 and 2019. Inclusion criteria were parents whose infants were admitted to the NICU for at least 5 days. Exclusion criteria were parents whose children were discharged within 5 days after admission or whose infants died in the NICU. Reliability was tested with Cronbach's alpha. The congruent validity was tested using Spearman's Rank correlation analysis, and the non-differential validity was tested using Cohen's d.

**Results:**

Parents of 619 infants discharged from the NICUs completed and returned the questionnaire. Most infants were male (*n* = 337, 54.4%) and infants' length of stay was a median of 21 days (IQR = 14–37). Mostly, mothers completed the questionnaire (*n* = 523, 84.5%). The Cronbach's alpha values of the five individual domains were between 0.67 and 0.95, and the alpha of the total questionnaire was.90, providing an adequate internal consistency. Congruent validity was measured by correlating the five domains with four standard satisfaction scales, documenting a weak correlation (*r*_*s*_ −0.025–0.327). Non-differential validity showed some significant effect size between four binary variables (mechanical ventilation, unplanned admission, admission after surgery, length of stay) four of the five domains.

**Conclusion:**

The Chinese version of EMPATHIC-30 questionnaire showed acceptable psychometric properties. This instrument might be considered a suitable instrument to measure parent satisfaction among Chinese parents whose infants are admitted to an NICU. Measuring parent satisfaction with this instrument might contribute to improving family-centered care initiatives in NICUs with Chinese parents.

## Introduction

Integrating patients' views into the development of patient-reported experience measures is important to capture the items that matter most to the patients (1, 2). Satisfaction instruments are often designed for hospital-wide patient satisfaction assessment and do not specifically address the various specialties within a hospital setting (3, 4). Within pediatrics, the family-centered care (FCC) approach is well developed, and parents are usually involved in the care of infants and children (5, 6). Consequently, satisfaction instruments for specific pediatric populations have been developed (7–9).

Family-centered care practices encourage parents to participate in the care and decisions of their children (10). Neonatal Intensive Care Unit (NICU) settings are environments with a complexity of treatments and care for critically ill infants. Admission to a NICU is often acute, and parents have little opportunity to shape expectations about the care for their infants. Still, they often face critical decisions and can experience stress during their admission (11–13). Standardized assessment of the experiences of parents in the care of their infants in the NICU is not formally assessed, which could potentially provide data for improving clinical practice.

Studies on patient satisfaction seem to be primarily aimed at assessing the care, with little regard for the overall experiences of perceived care (14). The value of satisfaction outcomes is the ability to benchmark practices that can lead to quality improvements. As such, a multidisciplinary approach is recommended, and the involvement of parents must be incorporated in these initiatives (15). The implementation of parent satisfaction outcomes in NICU settings provides a closer collaboration between parents and healthcare professionals that, ultimately, can improve the care of critically ill neonates and their parents (16).

In NICU, the parent satisfaction questionnaire, EMPATHIC-N, has been developed and used in several countries (17–19). This 57-item questionnaire has similarities with the short version of the EMPATHIC-30, developed in pediatric intensive care units (PICU) (20). This short version has been validated in PICU, NICU, and pediatric wards in Australia and showed good reliability and validity metrics (21). Therefore, the EMPATHIC-30 questionnaire can be considered a valuable instrument to measure parent satisfaction in pediatric and neonatal settings.

The EMPATHIC-30 questionnaire has been translated, culturally adapted, and validated among 101 parents in a Chinese PICU (22). To our knowledge, there are no validated questionnaires in Chinese to assess the satisfaction of parents of infants hospitalized in a NICU. Therefore, the aim of this study is to adapt and test the reliability and validity of the Chinese version of the EMPATHIC-30 questionnaire in NICU settings.

## Materials and Methods

### Setting

The setting of the study is the neonatal department at the stand-alone Hunan Children's Hospital, a regional tertiary center in Changsha, China. The department has four NICUs. NICU-1 is a 40-bed ward, admitting critically ill preterm infants, including mechanical ventilation; NICU-2 is a 60-bed ward, admitting stable term infants; NICU-3 is a 60-bed ward, admitting stable preterm infants; NICU-4 is a 40-bed ward, admitting critically term infants, including mechanical ventilation. The annual admission rate in 2018–2019 of all four NICUs was around 6,200 infants.

### Population and Recruitment

Inclusion criteria were parents whose infants were admitted to one of the four NICUs for at least 5 days. The exclusion criteria were parents whose children were discharged within 5 days after admission. The rationale for excluding these parents was the fact that many parents are unable to visit the NICU in these first days because the mothers were admitted to the maternity unit in another hospital or if mothers wished to follow the traditional 30-day confinement period. This Chinese tradition, zuò yuè zi (sitting the month), expects new mothers to stay home for a month to recover from childbirth. Also excluded were parents whose infants died in the NICU because these parents might have different views, and their discharge planning is different.

Non-probability sampling was used in our study. The sample size was calculated with the software G ^*^ Power 3.1 using the data of the development study of the EMPATHIC questionnaire (23). A sample size of 134 participants was estimated using the two-tailed test *priori* Wilcoxon–Mann–Whitney test for two independent groups, with an effect size of 0.5, an α of 0.05, and a power of 0.8. The data collection period was scheduled for 4 months, with the aim to recruit at least 134 parents from each individual participating NICU, with a total of 536 parents from all participating NICUs. Considering an estimated response rate between 30 and 40% in this type of research, we expect to recruit between 600–800 parents who will respond to the questionnaire. Data were collected between November 2018 and November 2019. Within this time, the total data collection time was 4 months as the study was paused for 8 months due to the recruitment of parents into an international study using other parent outcome measures.

Recruitment took place by the nurses in the NICUs. Parents were provided an invitation letter, consent form, and the EMPATHIC-30 Chinese questionnaire on the day of discharge. Once completed, the parents could deposit the completed questionnaire in specially dedicated return boxes in the NICUs. Unfortunately, the recruitment details of how many parents received the study invitation letter and the questionnaire were not completely recorded by the staff nurses. This limited us from calculating the response rate.

### Instrument

The EMPATHIC-30 has been translated and validated for the PICU at the Children's Hospital of Fudan University in Shanghai (22). A 10-step translation process included forward and backward translation followed by testing the instrument for cognitive equivalence by asking 10 parents. Debriefing among researchers took place during the process. The results of the validation testing were positive. The congruent validity of the instrument showed adequate correlation with four gold standard questions measuring overall satisfaction. Cronbach's alpha of the total score (30 items) was 0.96, and the split-half reliability coefficient of the total score was 0.879.

We accepted this version and reassessed the translation and cultural adaptation by consulting local parents to ensure the Chinese version can be adapted for parents whose infants are admitted to the NICU. The research team organized a parent consultation round with 10 mothers who have been involved in FCC in our NICUs. All mothers reported that they found the questionnaire easy to understand and that the answer option scale was easy to follow. We estimated that the average completion time of the questionnaire was around 15 min. After the consultation with the parents, few changes were made related to the wording of items to reflect the readability and understandability as suggested by the parents. The final version of the EMPATHIC-30 Chinese version for NICUs is provided in Supplementary Material 1.

### Data Analysis

Data analysis was performed using the Statistical Package for Social Scientists (SPSS, version 19.0). Descriptive statistics and non-parametric tests of difference were applied, and significance was set at *p* < 0.05.

The reliability of the questionnaire was assessed using Cronbach's alpha as a measure of internal consistency of the items within the five domains of the EMPATHIC-30 questionnaire and the total scale of the 30 items. Preferably, a Cronbach's alpha of >0.70 represents satisfactory reliability estimates (24, 25). The Spearman's Rank correlation for estimating the relationship between the statements on the domain level and four overall satisfaction-with-care questions (recommend NICU, return to NICU if needed, overall satisfaction with doctors, and overall satisfaction with nurses) was used for congruent validity. The non-differential validity was assessed by Cohen's *d* of the overall means of the individual domains and levels of four binary variables (mechanical ventilation, unplanned admission, admission after surgery, and length of stay). Means and standard deviations on the item level were calculated to determine the outcome of the satisfaction items.

### Ethics

Ethical approval of the study was granted by the Ethical Committee of Hunan Children's Hospital (Reference No. HCHLL-2018-34). All the participants were invited by means of an information letter, outlining the aim and methods of the study. Participation was voluntary and all questionnaires were anonymous. Written and signed consent forms were collected.

## Results

A total of 619 questionnaires were returned between November 2018 and November 2019. Mothers were the most frequent parents who completed the questionnaire (*n* = 523; 84.5%). The characteristics of infants and parents are presented in Table 1.

**Table 1:** Infant characteristics and parent characteristics (*n* = 619).

**Characteristic infants**	***n* (%)**
Gender; male	337 (54.4)
LoS NICU in days; median (P_25_-P_75_)	21 (14–37)^*^
Admission unplanned	238 (38.4)
Admission post-surgery	66 (10.7)
Mechanical ventilation	417 (67.4)
**Characteristics parents**	
**Who completed survey**	
Mother	523 (84.5)
Father	43 (6.9)
Both mother/father	47 (7.6)
Other	6 (1.0)
**Hometown parents;** ***n*** **(%)**	
Hunan province	555 (89.7)
Other provinces in China	64 (10.3)

* *median (P25-P75); LoS, length of stay; NICU, neonatal intensive care unit*.

Of the 30 individual items, 14 items (46.7%) scored a mean value <5.; all five items in the domain Information, three of the five items in the domain Organization, and all six items in the domain Parent Participation (Table 2). The item “Even during intensive procedures, we could always stay close to our child” scored the lowest among all the items (mean, 3.19; SD, 1.40). If this item was deleted from the domain Parent Participation, the alpha of the domain would increase from 0.84 to 0.88.

**Table 2:** Descriptive statistics and Cronbach's alpha of items and domains.

**Items and domains**	***n***	**mean (SD)**	**Cronbach's α**	**Cronbach's α if item deleted**
Domain information		4.71 (0.77)	0.93	
We had daily talks about our child's care and treatment with the: doctors	617	4.67 (0.87)		0.92
We had daily talks about our child's care and treatment with the: nurses	617	4.78 (0.86)		0.92
The doctor clearly informed us about the consequences of our child's treatment	617	4.68 (0.85)		0.90
We received clear information about the examinations and tests	617	4.75 (0.85)		0.92
We received understandable information about the effects of the drugs	617	4.69 (0.88)		0.92
Domain Care and Treatment		5.38 (0.49)	0.93	
The doctors and nurses worked closely together	611	5.44 (0.58)		0.91
We were well prepared for our child's discharge by the: doctors	611	5.40 (0.59)		0.91
We were well prepared for our child's discharge by the: nurses	611	5.39 (0.58)		0.92
The team was alert to the prevention and treatment of pain in our child	611	5.43 (0.60)		0.92
Our child's comfort was taken into account by the: doctors	611	5.39 (0.60)		0.92
Our child's comfort was taken into account by the: nurses	611	5.33 (0.59)		0.92
Every day we knew who was responsible for our child, regarding the: doctors	611	5.31 (0.66)		0.92
Every day we knew who was responsible for our child, regarding the: nurses	611	5.32 (0.63)		0.92
Domain Organization		4.93 (0.50)	0.67	
The team worked efficiently	619	5.56 (0.53)		0.68
The IC-unit could easily be reached by telephone	619	4.53 (0.83)		0.62
There was enough space around our child's bed	619	4.58 (0.90)		0.56
The IC-unit was clean	619	5.41 (0.64)		0.63
Noise in the IC-unit was muffled as good as possible	619	4.57 (0.83)		0.59
Domain Parent Participation		4.19 (0.80)	0.84	
During our stay the staff regularly asked for our experiences	619	4.09 (1.24)		0.79
We were actively involved in decision-making on care and treatment of our child	619	4.29 (0.90)		0.80
We were encouraged to stay close to our child	619	4.32 (1.04)		0.80
We had confidence in the: doctors	619	4.68 (0.87)		0.82
We had confidence in the: nurses	619	4.59 (0.89)		0.82
Even during intensive procedures, we could always stay close to our child	619	3.19 (1.40)		0.88
Domain Professional Attitude		5.37 (0.56)	0.95	
We received sympathy from the: doctors	544	5.33 (0.65)		0.93
We received sympathy from the: nurses	544	5.32 (0.64)		0.93
The team worked hygienically	544	5.42 (0.61)		0.94
The team respected the privacy of our child's and of us	544	5.38 (0.64)		0.94
The team showed respect for our child and for us	544	5.39 (0.63)		0.94
At admission we felt welcome	544	5.40 (0.63)		0.93
Total EMPATHIC-30-China		4.95 (0.41)	0.90	

The Cronbach's alpha values on the domain level were between 0.67 and 0.95. The domain Organization had the lowest alpha (0.67), while the Cronbach's alpha of the total scale was 0.90 (Table 2).

The congruent validity of the questionnaire was assessed by calculating the correlation between the answers of the five domains and the four standard satisfaction questions (recommend NICU, return to NICU if needed, overall satisfaction with doctors, and overall satisfaction with nurses). Overall, there was a weak correlation between the domains and the standard satisfaction scales (Table 3).

**Table 3:** Congruent validity domains of EMPATHIC-30-China with 4 general satisfaction items.

**Domains**	**Recommend NICU**	**Return to NICU if needed**	**Overall satisfaction doctors**	**Overall satisfaction nursing**
	***r_***s***_***	***r_***s***_***	***r_***s***_***	***r_***s***_***
Information	0.120^**^	0.050	0.255^**^	0.164^**^
Care and treatment	0.151^**^	0.050	0.249^**^	0.206^**^
Organization	−0.025	0.004	0.036	−0.024
Parent participation	0.146^**^	0.012	0.327^**^	0.223^**^
Profession attitude	0.165^**^	0.057	0.197^**^	0.098^*^

** *Correlation is significant at the 0.01 level (2-tailed)*.

* *Correlation is significant at the 0.05 level (2-tailed)*.

The non-differential validity, comparing the mean score in each domain between the binary characteristics of the infants, showed a large effect size in four of the domains related to mechanical ventilation of the infants and in two domains related to the length of stay. The parents whose infants had mechanical ventilation were more satisfied compared to the parents whose infants had no mechanical ventilation in the domains Information, Care and Treatment, Parent Participation, and Professional Attitude (Table 4). In the “unplanned admission” group, only a modest effect size in the domain Information was significant (Cohen's d, 0.25; *p* = 0.019). Although the cases in the variable “admission after surgery” were small, the effect size of the Cohen's *d* was moderate between 0.32 and 0.36, *p* < 0.02. In terms of the infants' length of stay, the parents in the <14-day group were less satisfied compared to the parents in the ≥14-day group in three domains of the questionnaire: Cohen's *d* between −0.47 and −0.23, *p* < 0.013 (Table 4).

**Table 4:** Non-differential validity between domains and characteristics.

**Domains**	**Yes**			**No**			**Cohen's *d***	***p***
	***n***	**mean**	**SD**	***n***	**mean**	**SD**		
**Mechanical ventilation**							
Information	416	4.92	0.60	202	4.29	0.89	0.89	<0.001
Care and treatment	412	5.51	0.42	200	5.11	0.53	0.87	<0.001
Organization	417	4.93	0.53	202	4.93	0.43	0.00	0.849
Parent participation	417	4.44	0.63	202	3.69	0.88	1.04	<0.001
Professional attitude	367	5.50	0.44	179	5.11	0.68	0.75	<0.001
**Unplanned admission**							
Information	237	4.83	0.76	381	4.64	0.77	0.25	0.019
Care and treatment	236	5.38	0.49	376	5.37	0.49	0.02	0.875
Organization	238	4.91	0.49	381	4.94	0.50	−0.05	0.749
Parent participation	238	4.27	0.79	381	4.15	0.81	0.15	0.087
Professional attitude	206	5.35	0.57	340	5.39	0.56	−0.07	0.379
**Admission after surgery**							
Information	66	4.97	0.71	552	4.68	0.77	0.38	0.012
Care and treatment	64	5.53	0.43	548	5.36	0.50	0.36	0.004
Organization	66	4.92	0.48	553	4.93	0.50	−0.02	0.829
Parent participation	66	4.42	0.71	553	4.17	0.81	0.32	0.018
Professional attitude	57	5.56	0.49	489	5.35	0.57	0.36	0.011
**Lengths of stay** <14 days^*^							
Information	171	4.59	0.92	447	4.76	0.70	−0.23	0.012
Care and treatment	171	5.21	0.53	441	5.44	0.46	−0.47	<0.001
Organization	171	4.96	0.40	448	4.92	0.53	0.09	0.455
Parent participation	171	4.05	0.89	448	4.25	0.76	−0.24	0.008
Professional attitude	149	5.22	0.62	397	5.43	0.53	−0.37	<0.001

*SD, standard deviation*.

* *<14 days was chosen to reflect the Q1 of the inter quartile rage (14–37) of the length of stay of the study cohort*.

## Discussion

The aim of the study was to validate the Chinese version of the EMPATHIC-30 among parents in NICU settings. The main findings of our validation demonstrated that the psychometric properties of this version are acceptable. The reliability estimates were very good for most domains, and the validity outcomes showed a weak correlation with other satisfaction scales. We chose this questionnaire because the EMPATHIC-30 questionnaire has been validated and used in several other countries and in NICU settings (26–29). Having a Chinese version of the EMPATHIC-30 for NICU settings allows us to benchmark parents' satisfaction data with national and international colleagues.

In our study, the items in the domain Parental Participation had the lowest mean scores. Although the Cronbach's alpha was good in this domain (.84), the possible reason is that the concept of FCC in our neonatal department is still in a developmental phase. Several FCC interventions have been implemented in our NICUs, demonstrating positive outcomes of infants and parents (30–32). However, we recognize that limitations in our FCC approach still exist, such as the exclusion of parents during medical rounds and not all staff are trained or prepared in daily communications with parents.

The findings of our study indicated that the parents rated 14 items below 5 on the 6-point Likert scale. Other similar studies using the EMAPTHIC-30 reported either similar low ratings or extremely high ratings. The study in South Africa reported high scores on most of the EMPATHIC-30 items in the PICU (33). Their lowest response scores were found in one item in the domain Information (information about effect of the drugs) and two items in the domain Parental Participation (regularly asked of our experiences and actively involved in decision). In contrast, the Turkish EMPATHIC-30 study in NICU settings reported no mean score >5 while using the same 6-point Likert scale. There were, however, no items with a mean score below 3 (26). The differences might indicate that a larger multinational cultural study is needed to identify factors in measuring parent satisfaction in different settings and different cultures. Recent reports have demonstrated the impact of the COVID-19 pandemic on family satisfaction in adult intensive care (34) or described the impact of the pandemic on zero separation of parents in the NICU (35). Our study period was before the COVID-19 pandemic. While the pandemic changed the FCC practices in our NICUs by limiting the visitation, further research is needed to explore the impact of COVID-19 on FCC practices. Reports have documented that the pandemic challenged the well-being of parents in the NICU, and stronger breastfeeding support was needed (36, 37).

There are several study limitations to address: First, our study was conducted in only one hospital setting, limiting the generalizability of the Chinese version of the EMPATHIC-30 in other Chinese NICUs or Chinese parents across the world. Secondly, we did not assess the test-retest validity of the instrument. Also, the time frame of the data collection might have influenced the results. During the study data collection period, we paused recruitment due to a family-integrated care study using other parent outcome measures. This study might have influenced NICU staff behaviors and attitudes on FCC that could have influenced the results of the parents after we resumed data collection. For example, this study included specific FCC education and training for doctors and nurses, which influenced the support to the parents. Thirdly, the questionnaires were mainly completed by the mothers. Fathers might have different opinions of the perceived care in the NICU, and it is suggested to maximize the efforts to include fathers in the care and support father so their voices are heard (13). Finally, we acknowledge the limitation of the EMPATHIC-30 questionnaire, not including allied health professionals. Most NICUs are expanding their teams with other health professionals, such as physiotherapists, psychologists, and social care workers. Evaluating their services should be recognized and could be the next step in further refining the questionnaire.

## Conclusion

The Chinese version of the EMPATHIC-30 performed acceptably on the reliability and validity testing among a large group of parents in the NICUs. The parents in our study had unique experiences and needs during their NICU stay, which have been translated in their responses to the items in the EMPATHIC-30-China questionnaire.

Our validated Chinese parent satisfaction questionnaire is timely and contributes to the international development of validated pediatric patient-reported outcome measures (PREMs) (38). This global expansion has been highlighted in a recent review, identifying 83 studies from 14 countries, describing pediatric PREMs (39). Most of the identified PREMs in this review include features that are related to patient- and family-centered care pratices, including shared decision-making and respecting the children and family values. Our EMPATHIC-30 questionnaire covers the FCC values, and the outcomes of our PREM can play a critical role in informing and transforming FCC practices in Chinese NICUs and beyond.

Further research is suggested to develop and test an online EMPATHIC-30 Chinese questionnaire that allows for ease of use for parents and ease of collecting parent satisfaction data on a national level (40). Such a system can support the implications for clinical practice to benchmark parent satisfaction data and learn from the data to improve clinical practice based on the experiences of parents.

## Data Availability Statement

The raw data supporting the conclusions of this article will be made available by the authors, without undue reservation.

## Ethics Statement

The studies involving human participants were reviewed and approved by Ethical Committee of Hunan Children's Hospital. The patients/participants provided their written informed consent to participate in this study.

## Author Contributions

YZ, RZ, X-rG, L-hZ, and JL contributed to design of the study. YZ and RZ contributed to the data collection. JL, YZ, and RZ contributed to the data analysis. YZ and JL drafted the first manuscript, and RZ, X-rG, and L-hZ provided revisions. All the authors contributed to the manuscript, read, and approved the submitted version.

## Funding

The study was partially supported by the Health and Family Planning Commission of Hunan Province (B2016031).

## Conflict of Interest

The authors declare that the research was conducted in the absence of any commercial or financial relationships that could be construed as a potential conflict of interest.

## Publisher's Note

All claims expressed in this article are solely those of the authors and do not necessarily represent those of their affiliated organizations, or those of the publisher, the editors and the reviewers. Any product that may be evaluated in this article, or claim that may be made by its manufacturer, is not guaranteed or endorsed by the publisher.
