# Supplementary material for: Validation of the Chinese Empowerment of Parents in the Intensive Care (EMPATHIC-30) Questionnaire Among Parents in Neonatal Intensive Care Units: A Prospective Cross-Sectional Study
Source: Front Pediatr. 2022 Mar 30;10:851291. doi: 10.3389/fped.2022.851291 (PMC9005953; doi:10.3389/fped.2022.851291)
Supplement: Supplementary file 1 [file Data_Sheet_1.PDF]

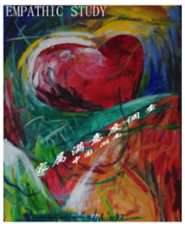

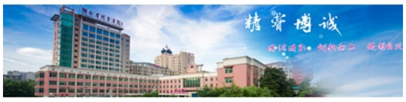

NICU 患儿父母的感受测评

亲爱的家长,监护人:

通过这份“NICU 患儿父母的感受测评”量表,我们希望知道在您孩子的监护期间您的感受如何。

这份问卷由一些关于您孩子和您自己的一些概况问题开始。接着将了解您关于入院流程、监护期间以及从新生儿重症监护室出院的一些情况。如果您同意条目，请偏向右边的方框作标记，如果不同意则偏向左边的方框作标记。

有些条目可能对您的情况不适用。如果是这样，请在“不需要”的方框内打钩。

示例:

	非常不同意					非常同意	不需要
每天都有人为我们提供一杯咖啡	☐	☐	☐	☐	☐	☐	☒
我们可以将我们的贵重物品上锁	☐	☐	☒	☐	☐	☐	☐
我们能够经常找到车位停车	☐	☐	☐	☐	☐	☒	☐

我们诚挚的希望您能在最后一页写下您的感受和建议,以便我们改进。

仅代表儿科重症监护室所有护士和医生向您的合作表示谢意。

庄严/张榕 医生

Jos Latour 教授

2018 EMPATHIC-30©

第一部分 您的概况

您孩子的姓名：__________	住院号：__________
您孩子的性别：	☐ 男<br>☐ 女
这份问卷由谁来完成?	☐ 母亲<br>☐ 父亲<br>☐ 父母一起<br>☐ 其他:____________
您的孩子多大?	________天 ________月 _________岁
您的孩子在重症监护室待了多久?	_________天 ________星期
孩子入住监护室是:	☐ 计划外的,没有预料<br>☐ 计划内的
孩子入住监护室是:	☐ 手术后<br>☐ 不是手术后
您的孩子接受机械通气吗?	☐ 是的 时间:__________<br>☐ 不是
您来自哪个省份?	_________

第二部分 您的感受

	非常不同意					非常同意	不需要
入住监护室时能有人很好的接待我们	☐	☐	☐	☐	☐	☐	☐
医生很清楚的告诉我们孩子的治疗结果	☐	☐	☐	☐	☐	☐	☐
我们可以非常清楚的接受到关于孩子检查和试验的信息	☐	☐	☐	☐	☐	☐	☐
我们可以接受到通俗易懂的关于药物疗效的信息	☐	☐	☐	☐	☐	☐	☐
医生和护士能紧密合作	☐	☐	☐	☐	☐	☐	☐
医护人员对我们孩子疼痛的预防和治疗很警觉	☐	☐	☐	☐	☐	☐	☐
我们能够主动参与我们孩子医疗照护方案的决定	☐	☐	☐	☐	☐	☐	☐
医护人员鼓励我们和我们的孩子近距离相处	☐	☐	☐	☐	☐	☐	☐
即使在操作时我们也能和我们的孩子在一起	☐	☐	☐	☐	☐	☐	☐
医护人员工作时注重卫生	☐	☐	☐	☐	☐	☐	☐
医护人员尊重我们和孩子的隐私	☐	☐	☐	☐	☐	☐	☐
重症监护病房很干净	☐	☐	☐	☐	☐	☐	☐
我们可以很容易电话联系到重症监护病房	☐	☐	☐	☐	☐	☐	☐
重症监护病房的噪音尽可能的低	☐	☐	☐	☐	☐	☐	☐
我们孩子的床边有足够的空间	☐	☐	☐	☐	☐	☐	☐
医疗护理团队能高效工作	☐	☐	☐	☐	☐	☐	☐
医护人员尊重孩子和我们	☐	☐	☐	☐	☐	☐	☐
在重症监护期间,工作人员定期询问我们的感受	☐	☐	☐	☐	☐	☐	☐

在入院时您的感受：

	非常<br>不同意					非常<br>同意	不需要
我们每天可以和_______讨论我们孩子医疗照护的问题：							
• 医生	☐	☐	☐	☐	☐	☐	☐
• 护士	☐	☐	☐	☐	☐	☐	☐
_______很重视我们孩子的舒适程度							
• 医生	☐	☐	☐	☐	☐	☐	☐
• 护士	☐	☐	☐	☐	☐	☐	☐
通过_______，我们每天可以知道谁负责我们的孩子							
• 医生	☐	☐	☐	☐	☐	☐	☐
• 护士	☐	☐	☐	☐	☐	☐	☐
我们对_______有信心							
• 医生	☐	☐	☐	☐	☐	☐	☐
• 护士	☐	☐	☐	☐	☐	☐	☐
我们从_______处可以获得同情							
• 医生	☐	☐	☐	☐	☐	☐	☐
• 护士	☐	☐	☐	☐	☐	☐	☐
_______很好的为我们准备出监护室事宜							
• 医生	☐	☐	☐	☐	☐	☐	☐
• 护士	☐	☐	☐	☐	☐	☐	☐

第三部分 总体感受

	非常<br>不同意					非常<br>同意	不需要
我们会向那些和我们有相似情形的人推荐此重症监护室	☐	☐	☐	☐	☐	☐	☐
如果再次面临相同的情形，我们还愿意来此重症监护室	☐	☐	☐	☐	☐	☐	☐

总体上您对我们的评价如何？

非常糟	1	2	3	4	5	6	7	8	9	10	非常好
• 医生	☐	☐	☐	☐	☐	☐	☐	☐	☐	☐	
• 护士	☐	☐	☐	☐	☐	☐	☐	☐	☐	☐	

在监护室治疗期间您的感受：

从重症监护室出院时您的感受：

您的总体感受：

我们很想从您的经历中有所学习,请随意的在下方写下您的感受。

（如果您需要更多的版面，请在反面继续写下您的感受）

仅代表新生儿重症监护室所有工作人员向您的合作表达诚挚的谢意。
